# Changes of serum aspergillus galactomannan during hematopoietic stem cell transplantation in children with prior invasive aspergillosis

**DOI:** 10.1186/s13052-016-0239-6

**Published:** 2016-03-09

**Authors:** Te-Fu Weng, Kang-Hsi Wu, Han-Ping Wu, Ching-Tien Peng, Yu-Hua Chao

**Affiliations:** Division of Pediatric Hematology-Oncology, Children’s Hospital, China Medical University, No. 2, Yuh-Der Road, Taichung, 404 Taiwan; School of post-baccalaureate Chinese medicine, College of Chinese Medicine, China Medical University, No. 2, Yuh-Der Road, Taichung, 404 Taiwan; Division of Pediatric General Medicine, Department of Pediatrics, Chang Gung Memorial Hospital, No. 5, Fuxing St., Guishan District Taoyuan City, 333 Taiwan; College of Medicine, Chang Gung University, No. 5, Fuxing St., Guishan District Taoyuan City, 333 Taiwan; Department of Biotechnology and Bioinformatics, Asia University, No. 2, Yuh-Der Road, Taichung, 404 Taiwan; Department of Pediatrics, Chung Shan Medical University Hospital, No. 110, Sec. 1, Chien-Kuo N. Road, Taichung, 402 Taiwan; School of Medicine, Chung Shan Medical University, No. 110, Sec. 1, Chien-Kuo N. Road, Taichung, 402 Taiwan

**Keywords:** Aspergillosis, Galactomannan, Hematopoietic stem cell transplantation

## Abstract

**Background:**

Invasive aspergillosis (IA) has recently increased and has a high mortality rate in immunocompromised patients. IA before hematopoietic stem cell transplantation (HSCT) is not uncommon, but how to cope with it is very tough. The serum aspergillus galactomannan antigen (GM) is a helpful marker for diagnosis of IA, and a serial follow-up of GM levels is important to evaluate the response of treatment. However, data on the changes of GM during HSCT are very limited.

**Case presentation:**

Patient 1 was a 2-year-old female with severe aplastic anemia. A typical lung lesion in the computed tomography of the chest with elevated GM levels was noted, and probable IA was diagnosed. After a combination treatment of voriconazole and caspofungin, the GM levels decreased. Although of significant improvement, the pulmonary lesion in the chest X-ray did not disappear before HSCT. The GM levels increased when she received the conditioning regimen during HSCT. The GM levels remained high during the use of steroids for the graft-versus-host disease and declined gradually after tapering off steroids and cyclosporine. Patient 2 was a 12-year-old female with severe aplastic anemia. Voriconazole was administered after the diagnosis of a probable IA. The pulmonary lesions in the chest X-ray disappeared before HSCT. The GM levels flared up during the administration of conditioning regimen and declined after neutrophil engraftment. At present, the two patients were cured of the disease without requiring surgical resection of their pulmonary IA.

**Conclusion:**

To our knowledge, this is the first report about the changes of GM during HSCT in patients with prior IA. With appropriate antifungal therapy and restoration of patient’s immunity, IA can be cured without surgical resection. Further studies are warranted.

## Background

Invasive fungal infections have been increasing and remain a major problem in immunocompromised patients [1–4]. Invasive aspergillosis (IA) is the most common invasive fungal infection and has a high morbidity and mortality rate, especially in patients undergoing hematopoietic stem cell transplantation (HSCT) [2]. The occurrence of IA prior to HSCT is not uncommon [5]. During the period of HSCT, it remains challenging to treat patients with prior IA due to the intensive chemotherapy or radiotherapy, profound and prolonged neutropenia, skin and mucosal damage, the use of aggressive immunosuppressants for graft-versus-host disease (GVHD), and GVHD itself.

The serum aspergillus galactomannan antigen (GM) is a helpful marker for diagnosing IA, and a serial follow-up of serum GM levels is important to evaluate the response of treatment [6]. However, data on the changes of GM during HSCT are very limited. Here, we reported the changes of serum GM levels in two children with severe aplastic anemia (SAA) and pulmonary IA prior to HSCT. They were successfully treated by antifungal therapy and allogeneic HSCT without surgical resection of their pulmonary lesions.

## Case presentation

**Fig. 1:**
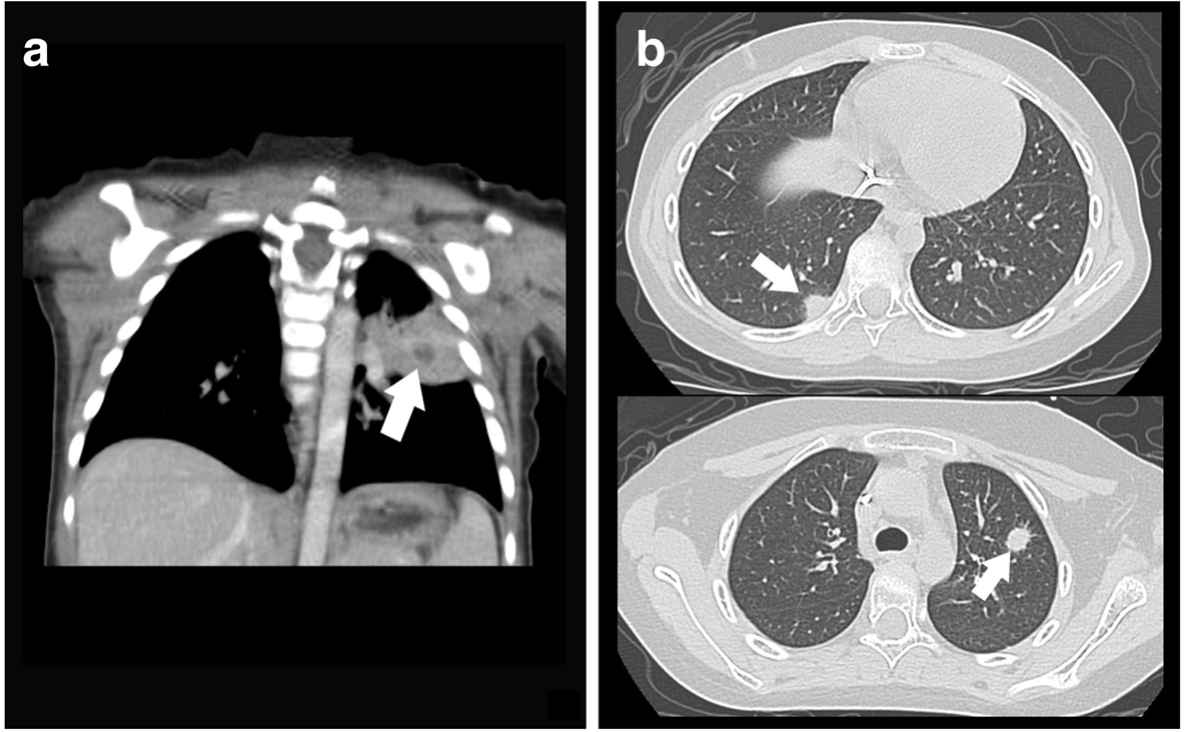
Computed tomography of the lungs. **a** In Patient 1, consolidation in the left lower lobe was noted (*white arrow*). **b** In Patient 2, there were nodules in the right middle and left upper lobes (*white arrows*)

**Fig. 2:**
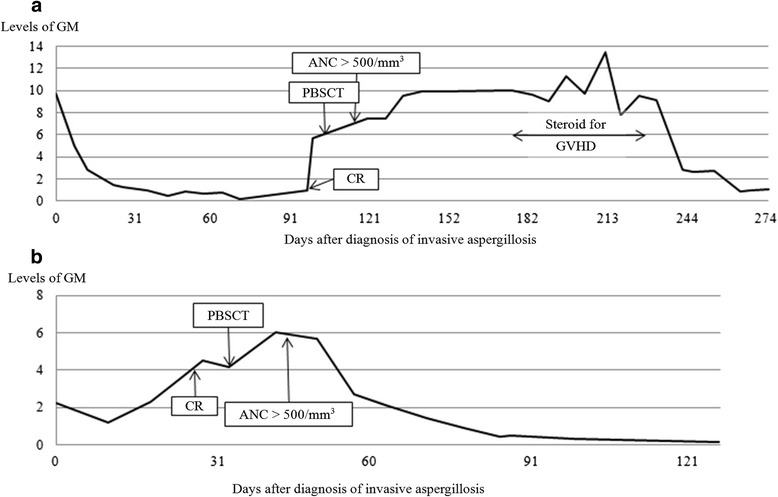
Changes of serum GM levels in Patient 1 (**a**) and Patient 2 (**b**) before and after PBSCT. *ANC* absolute neutrophil count, *GM* aspergillus galactomannan antigen, *GVHD* graft-versus-host disease, *PBSCT* peripheral blood stem cell transplantation, *CR* conditioning regimen

A 2-year-old girl (Patient 1) initially presented with severe diarrhea for 1 week. Peripheral blood showed severe pancytopenia, and profound hypocellularity of bone marrow without dysplastic changes and abnormal blasts were demonstrated pathologically. After the diagnosis of SAA was made, neutropenic fever with respiratory symptoms developed. Computed tomography (CT) of the lungs revealed consolidation in the left lower lobe (Fig. 1a). The serum GM was detected using enzyme-linked immunosorbent assay (ELISA) and the result was 9.75 (cut-off value <0.5). Therefore, a probable diagnosis of pulmonary IA was made. As the very low absolute neutrophil count, a combination of voriconazole and caspofungin was administered for their synergistic effects. The serum levels of GM continued to decrease, as shown in Fig. 2a. The pulmonary lesion in the chest X-ray was improved significantly but did not disappear before HSCT. The patient then received peripheral blood stem cell transplantation (PBSCT) from a 6/6 HLA-matched unrelated donor. The conditioning regimen consisted of cyclophosphamide, fludarabine, and rabbit anti-thymocyte globulin. Cyclosporine and methotrexate were used for GVHD prophylaxis. It was interesting to note that the serum GM levels elevated soon after the administration of conditioning regimen. Although high serum GM levels persisted, there was no progress of the pulmonary lesion and no new lesion was found, either. After a successful engraftment, a peak GM level of 13.42 was noted at the time of adding methylprednisolone for her GVHD. High GM levels (>7.0) persisted during the use of steroid for GVHD but declined gradually after tapering steroids and cyclosporine. One month later, caspofungin was discontinued and voriconazole was shifted to oral form. Eventually, the GM level declined to the normal range (<0.5) and CT did not reveal any pulmonary lesions, either. The patient had a full recovery of hematopoietic function without any pulmonary sequelae.

Patient 2, a 12-year-old girl, was diagnosed with SAA due to severe pancytopenia in the peripheral blood and extremely hypocellular bone marrow. She received immunosuppressive therapy with rabbit anti-thymocyte globulin and cyclosporine after diagnosis, but the treatment failed. Four months later, she suffered from neutropenic fever along with respiratory symptoms. Chest CT revealed nodules in the right middle lobe and left upper lobe (Fig. 1b) with the serum GM level of 2.24. A probable diagnosis of pulmonary IA was made, and voriconazole was given immediately. The use of voriconazole continued, but the GM levels increased to 3.57 (Fig. 2b). We performed PBSCT from a 6/6 HLA-matched unrelated donor for her, and the pulmonary lesions in the chest X-ray disappeared before HSCT. The conditioning regimen consisted of cyclophosphamide, fludarabine, and rabbit anti-thymocyte globulin. Cyclosporine and methotrexate were for GVHD prophylaxis. It was worth noting that the GM levels increased soon after the administration of conditioning regimen, to a peak level of 6.03 before recovery of neutrophil count. Then the GM levels decreased gradually, to the normal range 2 months after HSCT. She had a full recovery of hematopoietic function without pulmonary lesions in the follow-up CT scans. At present, these two patients are doing well with good quality of living. No medications, including antifungal agents, are needed.

## Discussion

SAA is a disorder of bone marrow failure, resulting in refractory and prolonged pancytopenia. Two major treatments for hematopoiesis recovery in SAA are immunosuppressive therapy and allogeneic HSCT [7]. In young patients with suitable donors, allogeneic HSCT is considered as the first choice. Infections are very common in patients with SAA because of their profound and prolonged neutropenia, even at diagnosis [8]. Mold infections increase in recent decades and Aspergillus is the most common mold of clinical importance [9, 10]. It is still extremely tough work to treat IA in patients with SAA because IA cannot be completely defeated in such hosts with prolonged and severe neutropenia. According to our experience here, we recommend performing HSCT as soon as possible for patients with SAA and prior IA as HSCT could improve neutropenia faster than immunosuppressive therapy in patients with SAA.

It is difficult to make a confirmative diagnosis of fungal infection. Unlike a bacterial infection, inflammatory markers in the blood usually are of limited clinical value [11, 12]. Serologic detection of circulating GM and 1,3-β-D glucan fungal biomarkers show promise for improving the diagnosis of IA. GM is a heat-stable heteropolysaccharide and is released during hyphal growth. With higher sensitivity and earlier positivity, ELISA is the preferred method over latex agglutination to detect GM [6]. Here, ELISA was performed to determine serum GM in these two children. There are several factors contributing to lower sensitivity and both false-negative and false-positive results in ELISA, including species of Aspergillus, presence of anti-Aspergillus antibodies, concomitant administration of certain β-lactam drugs, and cross-reactivity with other organisms containing GM. An optical density index ratio greater than 0.5 in serum is usually considered a positive result. In patients with hematological malignancies or HSCT recipients, the high specificity of ELISA but a variable sensitivity of 29–100 % has been demonstrated [6]. Molecular diagnostic assays, which have primarily targeted nucleic acid amplification through isothermal technique or polymerase chain reaction, are increasingly being interrogated as rapid and sensitive tests for detection of IA. However, a major limitation of their clinical use is a lack of standardization.

We speculated that IA could not be defeated and eradicated completely in patients with prolonged and severe neutropenia by merely using antifungal agents. In Patient 1, the pulmonary lesion in the chest X-ray was improved significantly after antifungal therapy, but it was still noted before HSCT. In Patient 2, even though there were no detectable lesions in the chest X-ray, the serum GM remained high levels before HSCT. It was a reasonable conjecture that IA existed persistently before HSCT in both patients. The prior existing IA flared up when immune function deteriorated, as the elevation of GM after the administration of conditioning regimen was found during HSCT. The serum GM elevated and persisted high until recovered from neutropenia. Moreover, the gradually decreased GM levels implicated the resolution of IA.

Surgical resection of pulmonary lesions can provide a definite diagnosis and can potentially eradicate a localized infection completely in patients with IA [13]. Surgical resection of a single pulmonary lesion prior to intensive chemotherapy or HSCT is an option of treatment in patients with IA [13]. However, the procedure is extremely difficult and dangerous to patients with SAA due to the risk of bleeding resulted from severe thrombocytopenia and the increased rate of infection derived from neutropenia. Here, both children with SAA and concurrent IA were successfully treated with antifungal agents followed by allogeneic HSCT to restore their immunity. No surgical intervention was performed before and after HSCT. Therefore, with the consideration of operation-related morbidities, surgical resection of pulmonary lesions may not be needed in this situation.

## Conclusion

With appropriate antifungal agents, it is still difficult to eradicate IA completely in patients with severe and prolonged neutropenia. Therefore, the prior existing IA flared up during HSCT could lead to elevated serum GM levels. After engraftment, the patients made a recovery from neutropenia, followed by an improvement of IA. The serum GM consequently decreased. To our knowledge, this is the first report about the changes of GM during HSCT in patients with prior IA. Further studies are warranted.

## Consent

Written informed consents were obtained from the patients’ legal guardians for publication of this report and accompanying images. A copy of the written consents is available for reviewing by the Editor-in-Chief of this journal.

## Abbreviations

IA: invasive aspergillosis
HSCT: hematopoietic stem cell transplantation
GVHD: graft-versus-host disease
GM: aspergillus galactomannan antigen
SAA: severe aplastic anemia
CT: computed tomography
ELISA: enzyme-linked immunosorbent assay
PBSCT: peripheral blood stem cell transplantation
